# Gingival tuberculosis

**DOI:** 10.4103/0972-124X.55836

**Published:** 2009

**Authors:** Sanjeev Jain, Bharti Vipin, Pankaj Khurana

**Affiliations:** *Professor, Department of Periodontology, GND Dental College and Res. Inst., Sunam, Punjab - 148 028, India*; 1 *Professor and Head, Department of Periodontology, Govt. Dental College and Hospital, Patiala, Punjab - 147 001, India*; 2 *Postgraduate Student, Department of Periodontology, Govt. Dental College and Hospital, Patiala, Punjab - 147 001, India*

**Keywords:** Gingiva, langhan cells, oral cavity, tuberculosis

## Abstract

Tuberculosis is a chronic specific granulomatous disease and a major cause of death in developing countries. The clinical presentation of tuberculosis lesions of oral cavity varies widely, including ulceration, diffuse inflammatory lesions, granulomas and fissures. Oral lesions usually appear as secondary to primary tuberculosis infection elsewhere, although primary infection of the oral mucosa by Mycobacterium tuberculosis has been described. We report a case of tuberculosis of gingiva, manifesting as gingival enlargement. Diagnosis was based on histopathological examination, complete blood count, X-ray chest and immunological investigations with detection of antibodies against Mycobacterium tuberculosis. Anti-tuberculous therapy was carried out for over six months. This case report emphasizes the need for dentists to include tuberculosis in the differential diagnosis of various types of gingival enlargements.

## INTRODUCTION

Tuberculosis is a specific granulomatous disease with a world-wide distribution. It remains a major health problem in most developing countries. India accounts for nearly one-third of the global burden of tuberculosis Every year approximately 2.2 million persons develop tuberculosis of which about one million are new-smear positive highly infection cases and about five lakh people die of tuberculosis every year.[1,2]

Occurrence of tuberculosis in the oral cavity is well documented in literature. Oral tuberculosis can be primary or secondary. Primary oral tuberculosis lesions are extremely rare and often occur in younger patients. It usually involves gingiva and is associated with regional lymphadenopathy. Secondary oral tuberculosis lesions are common and usually involve the tongue, followed by the palate, lips, buccal mucosa, gingiva and frenulum.[3–6]

Tuberculous lesions are seen as superficial ulcers, patches, indurate soft tissue lesions or even lesions within the jaw in form of osteomyelitis.[6,7] Here we present a rare case of gingival tuberculosis in a 38-year-old female.

## CASE REPORT

A 38-year-old female reported to the Department of Periodontology, Govt. Dental College and Hospital, Patiala with non- painful swelling of the gingiva, especially on the right upper side. The patient gave a history of enlargement of gingiva since past two years, which was gradually increasing in size with time. She had experienced loss of weight since the past four/five months. There was cough and weakness since 15 days.

Her medical history revealed no systemic problems except cough with expectoration since the past 15 days. The patient never visited a dentist in her lifetime and had no history of dental trauma or any surgery.

**Figure 1:**
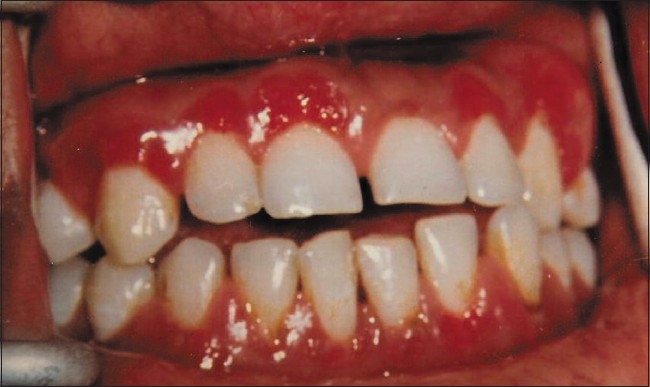
Fiery red, granular appearance of gingiva in upper and lower anterior area and upper posterior areas

Intra-oral examination showed gingival enlargement, especially in the upper and lower anterior labial and upper posterior buccal areas. The gingiva was fiery red, irregular, papillary, pebbled and granular in appearance [Figure 1]. The lesion was slightly painful on touch with spontaneous bleeding on provocation. It was uncommon in appearance with minimal of local deposits.

Extra-oral examination revealed no significant cervical lymphadenopathy. There was swelling of lips. Slight generalized swelling of the face was present more on the right upper side. Complete hemogram and IOPA X-rays were advised. Results of complete blood count were within normal limits, HIV test was negative and there was an elevated erythrocyte sedimentation rate (ESR). Provisionally, anti-allergic therapy was started.

**Figure 2:**
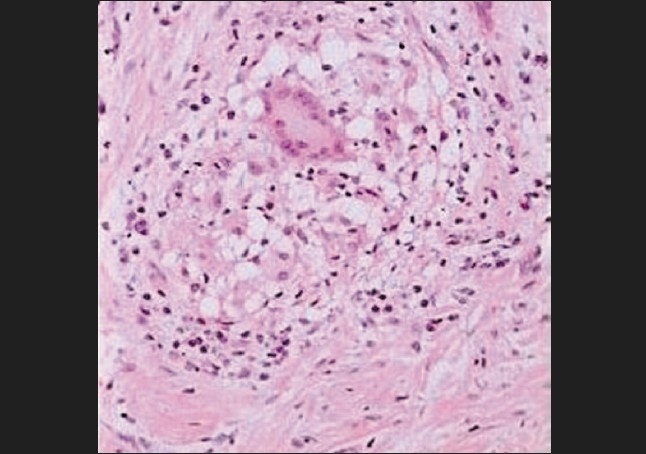
High magnification (40×) showing granulamatous lesion with langhan type giant cells, lymphocytes and epitheloid cells

An incisional biopsy was performed on the upper labial gingiva in relation to maxillary right central incisor. Histopathological examination showed papillomatous hyperplasia of the stratified squamous epithelium along with parakeratosis. In the sub-epithelial tissue, there were granulomas formed by epitheloid cells, langhans type giant cells, lymphocytes and some caseous necrosis [Figure 2]. The granulomas were present in upper part of the sub-epithelial tissue and even intra-epithelially. The features were suggestive of tuberculous granulamatous lesion.

The patient was advised X-ray chest and immunoglobins test for tuberculosis. Chest radiography revealed no abnormalities. An immunological test to detect antibodies against Mycobacterium tuberculosis in the patient's serum (ELISA) was positive. On the basis of history, ELISA and X-ray findings, the patient was confirmed as a case of gingival tuberculosis.

On consultation with the physician, anti-tubercular therapy was initiated with isoniazid (10 mg/kg of body weight), rifampicin (10-20 mg/kg of body weight) and pyrazinamide (10-20 mg/kg of body weight) for two months followed by isoniazid and rifampicin for the following four months. During the period, the patient was instructed not to undergo any surgical procedure within the oral cavity and was warned of transmitting the disease to others. However, conservative periodontal therapy, like scaling and root planing was carried out with minimal trauma to gingival and after consulting the physician in-charge.

## DISCUSSION

Tuberculosis remains the leading cause of death world-wide. South-East Asia carries a disproportionate 88 percent of the world's burden of tuberculosis. The vulnerability to tuberculosis in developing countries results from poverty, economic recession and malnutrition.[1,2]

There is general agreement that lesions of the oral mucosa are seldom primary, but rather secondary to pulmonary disease. Although, mechanism of inoculation has not been definitely established, it appears not likely that the organisms are carried in the sputum and enter the mucosal tissue through a small break in the surface. The organisms are likely to be carried to the oral tissues by a hematogenous route, to be deposited in the sub-mucosa and subsequently proliferate and ulcerate in the overlying mucosa.[4–6]

Tuberculosis has been recognized for many years as an occupational risk for health care workers, especially the dentists. The possibility that dentists may contract an infection from this contact with living tubercle bacilli in the mouths of patients who have oral tuberculosis or pulmonary tuberculosis is a problem of great clinical significance.[6]

It has also been shown that the presence of Mycobacterium tuberculosis in oral samples is almost universal in patients with tuberculosis. Viable acid-fast micro-organisms may be recovered from swabs or washings of oral cavities of tuberculosis patient. Furthermost, aerosol transmission of bacteria can occur during dental treatment such as ultrasonic scaling and use of air-turbine headpieces. Thus the diagnosis of oral tuberculosis is imperative in a dental set-up.

Tuberculosis of gingiva is a relatively rare entity.[8,9] It should be considered in the differential diagnosis, particularly in a non-healing lesion that does not respond to the usual therapy. Thus, a periodontist can contribute in early diagnosis and prompt treatment of such a highly prevalent and infectious disease as tuberculosis.[10]

### Differential diagnosis

- Gingival enlargements due to drugs
- Infections (bacterial, fungal and viral)
- Malignancy- leukemia
- Traumatic ulcer
- Squamous cell carcinoma

## CONCLUSION

A major concern for dentists and other health care workers, in light of re-emergence of the disease, is the risk of transmission of tuberculosis in the dental setting. Dentists are involved in the effort to control of tuberculosis through early detection and referral of patients to physicians for proper treatment and by developing and implementing appropriate infection control programs.
